# Extracts and Gleanings

**Published:** 1872-11

**Authors:** 


### INDIGESTION AND ITS MANAGEMENT.

BY BRADFORD S. THOMPSON, M. D.,
*Fellow of the New York Academy of Medicine, etc.*

READ BEFORE THE MEDICAL LIBRARY AND JOURNAL ASSOCIATION OF NEW YORK.

Dyspepsia, or indigestion, is an affection of very ordinary occurrence, and its management sometimes proves exceedingly perplexing to the practitioner. It is not necessary to enter minutely into its history, as it is accurately laid down in the various text-books and by writers on this subject.

*Symptoms.* Most commonly it is accompanied by nausea, vomiting, sour regurgitations, a sense of constriction about the throat, cardialgia, gastrodynia, pyrosis, constipation, chilliness, pallor, languor, an irregular pulse, and disturbed sleep. The power of digestion is not always uniform; on the contrary, it is sometimes very feeble, at other times preternaturally increased. The appetite may be depraved, the patient eating chalk, unripe fruit, etc. The disposition, if the disease continue any length of time, becomes very irritable. These are the ordinary symptoms of indigestion; but, besides these, there are occasionally others which are denominated *anomalous*. One of these is an acute pain in the chest and head, with no little perversion of vision. The effects are sometimes very extraordinary as regards the eyes. The writer had a case under his observation where everything appeared to the patient double; in another every object seemed inverted; and in another total blindness came on, which continued for twenty-one hours. The latter patient, a Conch woman, of Key West, Fla., aged forty-seven, was in the habit of eating prodigiously of a salad made from the indigestible conch, which abounds in that latitude.

Indigestion is also sometimes associated with a great degree of vertigo, and is liable to be confounded with apoplexy. Another anomalous symptom is palpitation of the heart, so violent as to be taken for an aneurism. The late Dr. Wistar, of the University of Pennsylvania, saw a lady from Charleston, S. C. who was suspected by the practitioners who first attended her of having an aneurism of the aorta. So great was the palpitation in this case that it lifted the bedclothes at every pulsation. By removing the indigestion with which she was suffering, at the end of three months she was ordered home entirely cured of the supposed aneurism.

The causes of dyspepsia are two-fold: 1. Such as act directly on the stomach; 2. Such as act through the medium of the general system. Among the first may be mentioned eating and drinking certain articles, such as strong tea or coffee, acrid substances, and gross, indigestible food. The immoderate use of opium and tobacco, in all their forms, often produce it. Those causes which operate through the medium of the general system are as follows: intense study, attention to business within doors, excessive venery, grief, and exposure to cold. These are the chief causes which produce *idiopathic* dyspepsia; but sometimes it is only *symptomatic*, arising from a diseased condition of the liver and its appendages.

Confessedly few diseases are more perplexing to the practitioner than the one under consideration. In detailing the best mode of treatment, the first indication, when acute indigestion presents itself from the presence of completely insoluble vegetable fiber, is to evacuate the contents of the stomach by emetics. By this procedure that viscus is not only relieved from noxious substances, but the organization is prepared for the operation of other remedies. Ipecacuanha and warm water and mustard, by common consent of practitioners, are to be employed for this purpose. They are sufficiently active, and besides exert a permanent impression against that condition of the system or stomach by which the disease is kept up. As cooperating in the same design are laxatives; but with regard to the selection some discrimination is requisite. All of the saline as well as the drastic purgatives are to be avoided. The only exception is rhubarb, which in very many cases may be advantageously administered. Indeed it seems to be particularly adapted to cases of this character by its tonic property. The only objection to this remedy is that it is apt to leave behind a tendency to constipation, which, however, is obviated by the combination of calcined magnesia or bicarbonate of soda.

The condition of the alimentary canal being rectified, tonics are then resorted to. Many of the vegetable bitters are very useful, particularly gentian, quassia, hop, and columbo. The hop and quassia are much employed with success, in many cases under our observation during the late civil war. Of all the tonics the hop, or its active principle, lupulin, is perhaps decidedly the best when dyspepsia arises from drunkenness or debauchery. Dr. James R. Wood's formula is to be recommended in the latter cases:

R.—Tinct. vlerian aromat.,
Lupulin, - - - - - - - - aa oz ij;
Tinct. cardamom., - - - - oz ss.
M. S.—A tea-spoonful as required.

In ordinary cases the mineral tonics are preferable, particlarly the chalybeate preparations. The carbonate of iron, in doses of ten grains, with a small proportion of the fluid extract of ginger, may be prescribed with utility; but the sulphate of iron, after repeated experience, is the most efficacious of the chalybeates, and should be given, in the form of pills, according to the subjoined formula.

| | | |
|---|---|---|
| R.—Ferri sulph., | - - - - | ʒj; |
| Acaciæ Arabicæ, | - - - | gr.xxx. |

This is to be made into thirty pills, one of which may be administered three or four times in twenty-four hours. Mr. McIntyre, of this city, a reliable druggist, prepares a nice preparation of cinchona and sulphate of iron, called elixir cinchona (ferrated).

Such are the remedies employed in many cases of indigestion; but there are symptoms occurring which demand a different mode of treatment. One of these is *Cardialgia*, vulgarly called *heart-burn.* In this affection emetics are to be avoided, the symptom being dependent on a superabundant acidity of the stomach. The remedies best adapted for this symptom are calcined magnesia, lime-water, milk, or any of the alkalies. The following prescription is very pleasant and serviceable:

| | | |
|---|---|---|
| R.—Potassæ subcarbonat., | - - | } aa ʒ ij; |
| Spirit. lavend. comp., | - - - | } |
| Sacchar. alb., | - - - - - | ʒj: |
| Tinct. opii, | - - - - | gtt.xxx. |
| Aquæ, | - - - - | ℥ iij ss. |

M. S.—A table-spoonful as often as the case demands.

In this state of derangement the stomach is frequently thrown into violent spasms called *gastrodynia.* Here we must have recourse to McMunn's elixir of opium, creosote, carbolic acid in doses of one to two drops in mucilage, or morphia hypodermically administered. In the case of the late Hon. Geo. M. Dallas a large goblet of milk always afforded relief. To prevent a recurrence of the paroxysm a few drops of turpentine in mucilage internally and a linseed poultice are very effective. A large sinapism is to be applied over the region of the stomach when the pain is violent and the preceding have failed. Sinapisms are useful in chronic as well as in acute affections of the stomach, and in the present instance they produce no less signal effects.

*Pyrosis.* This is a very extraordinary affection, and is endemical to some parts of the world, as Iceland, Norway, and

Greenland. It prevails also throughout the Highlands of Scotland, as well as in the western section of our country. Linnæus attributed it to smoked beef and a primitive mode of living; but this is not always the case, as it is frequently met with in the higher circles. Sometimes it has no ostensible cause. In this country it generally arises from an excess in eating or drinking. According to Chambers, "the liquid proceeds from the salivary glands."

In the treatment of pyrosis, or water-brash, the antacids are to be resorted to, particularly the saccharated solution of lime-water and milk—

R.—Liqoris calcis saccharati, . . f. dr. j–iv;
Lactis, ad., . . . . . f. oz. iv. M.

It may be well to remember that the addition of fifteen grains of bicarbonate of soda to the quart of milk not only prevents it from turning sour, but renders it more digestible. In this case much has been said of opium, but not much can be said in its praise. It sometimes allays spasm, and that is all. Much more may be expected from liquor potassæ and lime-water, bismuth, or carbolic acid. A single emetic is many times very useful, not only in evacuating, but also in changing the action of the stomach. Heberden, many years ago, advocated eight or ten grains of kino with half a gain of opium, and Tanner and others have since advocated this mode of procedure.

Indigestion, whether in its more simple form or associated with one or more symptoms which have been enumerated, is often exceedingly intractable, and sometimes will not yield to ordinary remedies. There is in many of these cases no organic affection of the stomach or disordered condition of the chylopoietic viscera, under these circumstances it appears to be established and riveted by long-continued habit. To meet this indication nothing is so efficacious as calomel in small doses; but it requires courage to call it by name in these days of eclecticism and homeopathy.

Dr. Chambers's favorite tonic in chronic indigestion is quinine in two-grain doses, in lemon-juice sufficient to dissolve it, and diluted with water to a convenient bulk. Its action seems to be principally on the mucus membrane of the mouth, œsophagus, and stomach, which it astringes and tones up to a healthy state, retaining the secretion of mucus, and making the special secretions more active. To quinine he usually adds from one twenty-fourth to one twentieth of a gain of hydrochlorate of strychnia, unless there are some contraindications to its use. In children especially the use of Baudault's pepsin wine can be highly recommended. This is prepared, from pure pepsin, according to the formula of Dr. Corvisart, and is very palatable. Each dose possesses fully the digestive power of fifteen grains of the powder. This preparation, I state with much confidence, is superior to all other preparations of pepsin in use. It should be given immediately before a meal.

To complete the description of indigestion attention is called to some of its uncommon forms. One of these is frequently met with, but is only mentioned by one or two authors. It is a slow, *chronic inflammation of the stomach*, and was formerly attributed to intemperance by Dr. Chapman, of Philadelphia; but, it having been observed in persons strictly tomperate, this opinion is no longer entertained. It is distinguished by pain at the pit of the stomach, quick pulse, dry cough, a slow fever, and wasting of the body. It resembles very much pulmonary tuberculosis, and may be confounded with that disease. Small doses of ipecacuanha may be beneficially employed, but so minute as not to create nausea. Given in this way it acts, as the older writers would say, as an alterative; changing the morbid condition of the stomach, and restoring its natural and healthy character. This remedy was first advocated in affections of the stomach, about a century ago, by Dormitian, a French writer.

Another variety of this disease is that which by the *inordinate use of spiritous liquors.* This form is marked by great irritability of the stomach, flatulence, vomiting, the food being rejected at once or patially digested. These symptoms may be very suddenly removed, by the use of opium or morphia, tincture of valerian, dry sherry, and the Hungarian wines. In this form, when death is occasioned, dissection reveals a considerable organic affection of the stomach, having a smooth, leaden hue, with the mucous coat destroyed by the continued application of alcohol to its surface.

In these violent cases there is always great gastric distress and vomiting. By the use of proper remedies the stomach generally recovers its tone and healthy action; but to establish a permanent cure the habits of the individual must be changed, and great attention paid to a proper course of diet and all habits of life.

As the stomach is the immediate seat of the disease and the receptacle for the food, nothing appears to be more indicated than the propriety of selecting those articles which are the lest disagreeable to that organ. Compared with every other course of living, a milk diet is decidedly to be preferred. It will often of itself effect a cure, and there is scarcely a symptom which it will not relieve. In gastrodynia, cardialgia, and pyrosis it is eminently useful. On account of certain idiosyncrasies it sometimes cannot be employed, but it is believed such cases are rare.

It has been objected to milk that when rejected from the stomach it sometimes appears coagulated; but even in the most healthy stomach it coagulates as soon as the gastric juice begins to act upon it. This occurs in the first stage of digestion. Indeed without undergoing this alteration digestion and assimilation could not be carried on. This cannot therefore constitute a valid objection to the use of milk. At first the patient may imagine that he does not derive benefit from it; on the contrary temporary oppression. It should not be abandoned too hastily, for this will soon be overcome by a continuation in its use. Cordovin long ago remarked that when milk at first disagrees with the stomach it is a certain indication that the patient demands a milk diet. Cases nevertheless may occur where, from the prejudics of the patient or from real injury, its employmet would not be advisable. Under such circumstances there should be no hesitancy iu substituting chocolate. It is of much importance that this be properly prepared, and the best mode is the following: first boil the chocolate in water and set it aside to cool, then skim, reboil, bring to the table, and pour it on sugar and cream. In this way the feculent and oily matter is avoided, and it is rendered exceedingly digestable. Neither tea nor coffee should be allowed. For dinner, beef-essence, mutton, chicken, turkey, white-fish, or oysters may be recommended. Neither pork, ham, veal, duck, or any of the salt meats are ever admissible in this affection. Soups are generally distressing and injurious, especially if badly made. The proper vegetables for the patient are roasted potatoes, boiled rice, tomatoes and asparagus. Every species of desert ought to be carefully avoided. The bread used should be toasted, or the Boston cracker substituted. Under no circumstances should butter be used.

Dr. Thos. King Chambers suggests the following ladder of meat diet: "Whey, milk and lime-water, milk and water, plain milk, milk and sago, milky rice-pudding, beef-tea, plain mutton-broth or chicken-broth, Scotch broth, turtle-soup, sweet-bread, boiled fish, especially water souchy, boiled partridge or boiled chicken, mutton chop, gridled in the air and without fat, roast joint of mutton. Roast joint of mutton is the promised land of the convalescent. When he arrives at that it is a matter of time and strengthening the stomach by occasional tonics for him to come to the digestion of anything within the scope of his birthright; and even if he has to stop at roast joint of mutton he will stop at a very good thing."

In regulating the diet impress upon the patient the necessity of observing the subjoined rules:

1. Enjoin frequent and regular eating in the majority of cases. It was a remark of Sir William Temple "that the stomach is like a school-boy; if idle, always in mischief." The deduction drawn from this is to keep the stomach moderately employed.

2. Let the diet be simple, always consisting exclusively of one article.

3. Drink little or nothing while eating.

4. Exercise should not be permitted directly after eating.

In many cases a voracious appetite attends this affection; but in the majority of cases there is very little inclination to eat; and under these circumstances it will not be amiss to attend to the following particulars for the purpose of exciting the appetite:

1. Do not let the patient know what he is to eat.

2. The food should always be cold; when hot the odor sometimes destroys the appetite.

3. The dishes should always be small, for nothing is more distressing to a patient with a delicate stomach than a large dish of meat set before him.

These circumstances, though apparently trivial in their character, are very important and deserve recollection.

In regard to drinks, water, as a general thing, is to be preferred to any other. In some cases good porter answers exeeedingly well, yet it often proves injurious. Beer, port wine, and undiluted liquors should never be given, as they almost always sour on the stomach and disagree with the patient. Pure old Jamaica rum, well diluted, when the patient has been accustomed to the use of distilled liquors, is sometimes admissible in very small quantities; but the physician should be very careful that it be given sparingly.

In some instances much advantage may be derived from directing remedies to the system generally. For this purpose the warm, sea, or Turkish bath should be employed once or twice a week. The cold bath, in many cases, is equally useful. Tonic remedies are also often required.

Exercise, particularly on horseback, constitutes on important part of the treatment, which will alone sometimes accomplish a cure. Walking is also beneficial. You may also permit and even encourage the patient to visit some of the watering-places. By this means, as the saying is, "you kill two birds with one stone." For, independent of the pleasure derived from the jaunt, the patient will be benefited by the exercise, and the mineral springs, by their tonic properties, have a salutary influence. Many persons have been cured by visiting Saratoga and using the waters there in moderation.

Every practitioner is well acquainted with the great sympathy which subsists between the alimentary canal and the surface of the body. The propriety therefore of keeping the body warm is very evident. Too little attention is paid to clothing. It being of the utmost importance in preserving the warmth of the surface, flannel should be worn next the skin. The utility of this practice in affections of the bowels is well known, and it is no less useful in chronic diseases of the stomach.

But what will all that has been said avail if the remote causes are not avoided, and if the patient does not renounce those habits and pursuits which, either directly or indirectly, have a tendency to keep up the disease? If the patient be intemperate, let him return to a regular and sober life. If luxurious, let him reform his habits. If indolent, he should be roused early to enjoy the salubrity of the morning atmosphere and awakened to enterprise. If studious, let him abandon his midnight lamp. The patient never should be allowed to despair. On the contrary, always encourage him in an expectation of recovery, arousing his hope, and encouraging his faith in the future; and there is hardly a case which may not ultimately be relieved or entirely cured.—*American Practitioner*, *July*, 1872.

---

HYDROCELE CURED BY CARBOLIC-ACID INJECTIONS.—D. P. E. Sandidge, of Edmonton, Ky., writes: "Having seen nothing during the carbolic-acid mania of the use of this many-sided remedy in hydrocele, I send the following note: In March, 1868, Mr. W., aged sixty-five years, observed the tunica vaginalis of the right side to be greatly distended with a fluid. There was some also in the left side. Both tumors were punctured, and the fluid withdrawn. That on the right side was darkish; that on the left was perfectly limpid. The sacs were now injected with the tincture of iodine, which was allowed to remain, and in due time the case was discharged cured; but the tumors gradually reappeared, and in April, 1871, had acquired their former size. They were again emptied, the fluid in the right side being darker and thicker than before. I now threw into the vaginal tunic of this side, instead of the iodine, two drachms of Calvert's solution, No. 5, with a small quantity of water added, but repeated the iodine on the left side. The patient suffered some at the time, and complained afterward of fever in the right, with frequent erections. The left side gave no trouble. A brisk purge or two, rest and diet, with cooling lotions to the parts, straightened out matters, and in ten days he was dismissed. The right testicle and the right side of the scrotum was considerably retracted. The left testicle hung as usual. In January, 1872, the fluid had reaccumulated on the left side, the right being unaffected. The tumor was opened, contents evacuated, and carbolic-acid injected as in August. The patient experienced much the same pain, etc., and had the same after-treatment. The testicle and scrotum of that side retracted as the right had previously done. Six months after the operation there was no sign of a return of the disease."—*American Practitioner*, *July* '72.

---

Dr. Wm. Richardson's Treatment of Diabetes.—Dr. Richardson (*ibid*) was himself attacked ten years ago with diabetes. After a prolonged trial of the most approved remedies he was fortunate enough to hit upon a plan of treatment by which he has been cured, and by which also other diabetic patients have been much benefited. The essential features of this plan are the employment of regular and steady exercise, ablution of the skin daily with soap and water, the use of a bath, containing a tablespoonful of carbonate of soda, twice in the week; exposure of the body as far as practicable to sunlight, and the continuous use of iron, which he uses in the form of tincture of the perchloride in four or five drop doses, with one or two drops of tincture nux vomica and eight or ten grains of chlorate of potash three times daily. He is an advocate of a restricted diet; but when the plan of treatment which he suggests is carried out fully he finds that a considerable amount of relaxation as regards food is not injurious. He regards the sudden adoption of a very restricted diet as likely to prove highly prejudicial. Dr. Richardson's present dietary is sufficiently liberal, and, besides meat, includes brown bread, with plenty of fresh butter, macaroni, and rice, potatoes sparingly, and occasionally a little dry fruit. Even a few glasses of champagne occasionally he does not find at all injurious.

---

Treatment of Hæmorrhoids.—Hæmorrhoids, connected with prolapsus, were successfully operated upon by the application of nitric acid some thirty years ago by Dr. Houston, of Dublin. The method found great favor at the time, but has been somewhat displaced by the ligature, and the section followed by the actual cautery. Billroth, of Vienna, has of late revived Houston's operation, and reports excellent results, especially in those cases where frequent and dangerous hemoarhage had occurred.—*Lancet*, June 1, 1872.

### PART II.

### Abridged Extracts and Gleanings from our Exchanges

*MULTUM IN PARVO.*

Spinal Irritation.—Dr. W. H. Phillips, (Medical and Surgical Reporter, Sept. 7, 1872), speaking of the treatment of spinal irritation, remarks:

"There are three leading indications to be filled in the treatment of this disease.

1st. To remove the cause.

2d. To restore the general tone of the system.

3d. To improve the circulation and nutrition of the cord at the diseased part.

It is of the most essential importance to discover the real cause, in order that measures for its entire removal may be set on foot as a preliminary. If the patient gives a history of hemorrhages, lucorrhœa, prolonged lactation, or any other exhausting discharge, measures must be adopted for their special treatment and removal, in order that this exhausting flow of the elements of vitality may be directed to their proper channels.

In the fulfillment of the first indication, namely, the removal of the causes, we enjoin rest; not absolute, but in a recreative sense; the strict avoidance of fatigue or excitement.

The second indication is best accomplished by the use of such general tonics as have proven most efficacious in our hands. Quinine, iron and cod-liver oil, are confessedly at the head of this class of remedies.

In addition to this class of direct tonics, all who have had opportunity for observation, agree that alcoholic stimulants are among the most efficient means of treatment at our command.

In the accomplishment of the third indication somewhat greater skill and care is required. To determine a greater flow of blood to the spinal cord, and thereby promote its nutrition, a number of special remedies have been used with success. Of these strychnine, phosphorus and zinc, are worthy of special mention.

I have used with great satisfaction a combination of these remedies in the form of the phosphide of zinc in combination with nux vomica.

There is another agent which I have used with very gratifying results, namely, the constant galvanic current. It is claimed by experts that this agent is capable of producing an increased or diminished supply of blood to the cord at will, according to the direction of the current used.

The next means for the accomplishment of this object is by small and frequently repeated blisters.

This outline embraces the chief remedies at our command for the treatment of this peculiar disease."

Rules for Cold Bathing.—Dr. Walton, in his recent work on Mineral Springs, gives the following

RULES.

1. The most favorable time of day for taking a cold bath is on rising in the morning, or about noon.

2. The stomach should be empty when the bath is taken.

3. Exercise moderately before entering the bath, and while in the bath; but the body must not be overheated, on going into the water.

4. A cold bath should not be taken when fatigued.

5. The duration of a cold bath should not exceed five minutes.

6. The cold bath should be succeeded by friction of the surface with a coarse towel or flesh-brush.

7. If the cold bath is not followed by reaction, the duration has been too long, or cold bathing is not fitted for the individual.

8. The cold bath is not adapted to feeble or aged persons or infants.

9. Persons whose extremities or skin are usually cold should not use the cold bath.

10. Persons affected with organic disease of the heart should not take the cold bath.—*Medical and Surgical Reporter.*

Ileus Successfully Treated by Electricity.—Dr. Bogdan, reports in the "Wiener Medizinische Presse," March 10, 1872, a case of ileus successfully treated by electro-magnetism, after the more usual remedies had failed to afford relief.—*Ex.*

Lithotrity in Children.—Dr. Guersant (Medical News and Library) remarks that his own experience and of all those who have performed this operation is favorable to the employment at all ages, even in children fifteen or eighteen months old, without regard to sex. Lithotrity is always applicable to the youngest children when—First, the calculus is of but small size, being not more than three-fifths to two-thirds of an inch in diameter, and the surgeon is enabled to operate several times—three times at the most. Second, when the general health is good, and the bladder appears to be in good condition and exempt from purulent catarrh. Third, when, above all, there are not more than one or two calculi. Lithotrity is, on the contrary, inapplicable—First. When the calculus is too large (three-quarters of an inch or more). Second. When it is adherent. Third. When it is very hard and mulberry-shaped.

A Mode of Operating for Radical Cure of Varicocele.—Dr. H. B. Davison, San Francisco, (Pacific Medical and Surgical Journal), has adopted a new mode of operating for the radical cure of varicocele, for which he claims three great advantages over any other means:

First. By perforating only one wall of the scrotum, less pain, less inflammation, and less risk of adhesion of the wounded sac and spermatic cord.

Second. By placing the patient in a recumbent posture when the operation is being performed, so that no blood may be enclosed in that portion of the vein cut off from the circulation, the resultant inflammation will be much less, and the testicle will not swell so much, and absorption will be accomplished in much less time.

Third. By removing the ligature before it cuts through the vein the risk of phlebitis is lessened, and the patient is enabled to resume his ordinary duties much sooner.

Those who have been operated on have no return of the disease, and it would require a very close examination of the parts to discover that any operation had been performed. In one case the patient had been wearing a suspensory bandage for over twenty years, and the left testicle was much atrophied. It is now about sixteen months since the operation, and the patient has a corresponding increase of sexual power.

Vomiting and Salivation—Remaining from intermittent fever were cured by tincture of Iodine, ten drops thrice a day. Two favorable cases are reported, where quinine and arsenic had failed.—*Memorab.—Indiana Medical Journal.*

The Use of Acetate of Lead in Cavities of the Lungs.— Dr. Traube, in the Berl. Med. Gesell., says there is a kind of ulceration of the lung, when only one or a few cavities are present, and only inflammation exists in other parts of the lung; such cases of circumscribed ulceration are for the most part curable, and this accounts for Skoda's cases of cure by turpentine inhalation. But such inhalations had the disadvantage of increasing the inflammation around the cavities, sometimes to a fatal extent. Traube on this account uses the remedy which was formerly recommended, acetate of lead, in inflammation of the lung in the dose of 0.06 grammes every two hours, and in some cases also local blood-letting; if the expectoration and fever cease a litte, tannic acid is then given, and a majority of the patients are cured

Lead has, besides its antiseptic property, which it shares with the astringents, only an antiphlogistic, but no antifebrile effect, as has been seen by Traube in cases of lead colic by the thermometer. It must, therefore, if we would strive to master the intensity of the fever, be accompanied by a febrifuge, and digitalis is one of the best. In cases of ulceration of the lungs this is not necessary, but it is useful in cheesy pneumonia, when the attempt should be made to master the inflammatory process and make the products be absorbed; in such cases the dose must be somewhat greater. A fortifying line of therapeutics can only be indicated when cavities are being formed; even in cases of cheesy pneumonia, which are cured, an over powerful nourishment may do good, and may lead to abmormal pressure in the pulmonary arteries, and the formation of small arteries therein, so that the patient may die suffocated by the rupture of some of these.

Acetate of lead is also useful in some cases of catarrh of the bladder, and is used by Traube locally by injections of 6, 12, 18 centigrammes in 180 grammes of water, twice or thrice daily in some cases.

Digitalis and acetate of lead are given in the form of powder when combined, as they destroy each other when fluid.—*Doctor*.

Morphia and Chloral in Combination. By R. H. Fisher, M.D., of Kennekuk, Kansas.—For the last year I have used morphia and chloral combined, with the best results. A single example will illustrate this. Last winter I was called up late one night to see a fellow practitioner suffering intense agony from rheumatism. His wife had already given him about a grain of morphia without relief. I immediately administered ten grains of chloral, and in ten minutes he was free from pain.

The Treatment of Melancholy.—Dr. Mendel, of Paukow, (Memorabilien, July, 1872,) says that it is a mistake to treat melancholy by means of concerts, society, theatres, and the like. It is caused by hyperæsthesia of the brain, and requires rest from all kinds of excitement. The patient should reside in some very quiet and retired country place. This often produces a rapid cure, and sleep returns to the patient, who has long suffered from insomnia. Warm baths are also to be recommended. A great proportion of such cases get well spontaneously, and do not require any medicine internally. The use of morphia subcutaneously injected, however, is sometimes indicated. Dr. Mendel speaks very highly also of chloral when a temporary sedative is required, since it is much quicker in its operation than morphia injection. The author has found in many cases of chronic melancholy thickening of the pia mater, combined with hyperæmia of the brain. In many cases where morphia injection is useful, symptoms of pressure have been remarked by the author, such as slight paresis of the facial nerve, deviation of the tongue, of the uvula and difference in the size of the pupils.—*Doctor.*

Red Precipitate Ointment.—The United States Pharmacopœia directs lard ointment for making unguentum hydrargyri oxidi rubri, but having had frequent difficulty in preserving the beautiful reddish color of the ointment, I was induced a few years ago to institute a series of experiments to obtain a more reliable preparation. After many experiments I became convinced that a compound of castor oil and white wax would be just what I wanted. The following is the formula I have adopted:

R.—Hydrarg. Oxid. Rub. sub. pulv., oz. i-;
Olei Ricini, oz vi.;
Ceræ Albæ, oz. ii.
Misce l. a.

This ointment will preserve its beautiful reddish color and proper consistence for years without change.—*Ex.*

Temperature in Diseases.—Dr. Alvarenga, of Lisbon, (La France Med., August 10,) mentions that the maximum temperature noticed in man has been 44 deg-75 deg. C., in a case of tetanus; the minimum was observed by Hardy 22 deg. C., in a case of sclerema; but it is rare to see the thermometer higher than 48 deg. C., or lower than 32 deg. C. The work of Dr. Alvarenga is a credit to Portugal.—*Doctor.*

Treatment of so-called Hysterical Joint.—Dr. Eshmarch, (Memorabilien, July, 1872,) says that in the treatment of hysterical joints, great attention must be paid to the general health. Blisters, etc., are worse than useless, and so are all lowering remedies. Again, absolute rest of the limb is useless in such cases, and, indeed, sometimes does harm. Passive motion of the limb should first be tried, in spite of the appearance of suffering; and next rather energetic movements of the limb must be tried. In some cases chloroform should be used and the limb moved to prove that there are no morbid adhesions. As soon as the muscles are quite put to sleep, the obstinate contraction disappears and the movement of the joint becomes free. This should be only done once, as the patients easily begin to desire to have chloroform given to them. No operative precedures are indicated in such cases. Cutting of the nerve, too, is useless. Energetic kneading of the limb is to be avoided. Cold douches are often useful locally applied, with rubbing of the limb, and passive and active motion. Iron and nourishing diet are the two chief remedies, combined with the water-cure and sea-bathing. The so-called nervine tonics and anti-spasmodics, such as assafœtida, valerian, etc., appear to the author of but little use; but the use of arsenic is sometimes indicated. Treatment of the mind occasionally effects a cure by itself.—*Doctor.*

Cauterizing Venereal Sores.—Dr. J. Rogers, of this city, believes the following to be a decided improvement on the present method of cauterizing venereal sores, as it causes little or no pain, and seems to give quite as favorable results. First, saturate the sore with a solution of carbolic acid (gr. xx. to aq. oz. j.), using a brush or atomizer, the latter being preferable; then touch the part with pure carbolic acid, followed by pure nitric acid. The above has given him entire satisfaction for nine or ten months past, and, he no doubt, will be valued by those who choose to try it.—*Ex.*

Surgical Treatment of Anasarca.—In a severe case of anasarca, Dr. Wolff (Berlin Klin. Wochenschr., No. 141, 1872) introduced into the skin a number of fine canulæ, such as are used for subcutaneous injection, and left them there. Through twenty-five canulæ thus used, twenty quarts of fluid were discharged in three days. The fluid was carried away in elastic tubes into a vessel near the bed. No inflammation of the punctures, nor any other unpleasant results followed the operation.—*Boston Med. and Surg. Jour.*, June 28, 1872.

Anodyne Alterant.—

| | | |
|---|---|---|
| R—Ext. Butternut, | . . . . | dr. xiv. |
| Ext. Hyoscyannus, | . . . . | dr. ii. |
| Oil Sassafras, | . . . . | dr. i. |
| Bicarb. Soda, | . . . . . | dr. iv. |
| Sim. Syrup, | . . . . . | oz. xvi. |

Dissolve extracts, oil and soda in the syrup by a gentle heat. Dose, from teaspoonful to tablespoonful three times daily, or oftener if required.

The above was a favorite prescription, much used by the late Dr. Thompson, of Tennessee, well calculated to allay nervous excitement, ease pain, procure rest, neutralize acidity and regulate the stomach and bowels without debility. Will meet the indications, in most forms, of dyspepsia, by giving a teaspoonful after each meal with perhaps a tablespoonful at bed-time to keep up a proper action of the bowels.

The above will be found valuable in the countless ills of pregnancy, and is to all intents *the retired mother's relief.* In cases of habitual abortion, if used prior to anything like considerable uterine expulsion, will rarely fail of the desired end.

Lastly, we find it very efficient in quieting inordinate after-pains, and at the same time gently opening the bowels when used for two or three days subsequent to delivery. We say to our brother country practitioners, prepare a bottle and try it.

George D. Hodge, M. D.

Holly Springs, Arkansas.

Antidote for Arsenic.—Professor Carles, (Medical and Surgical Journal) found that sugar rendered the arsenite of magnesia soluble, and thus counteracted the desirable reaction of the magnesia. It was also noticed that with the exception of arsenic, the sugar did increase the antidotal reaction of the magnesia. A mixture of 2½ drachms of magnesia, 5 to 6 drachms of sugar, and 3 ounces of boiling water, is represented as being a very good composition of the antidote.

In Cases of Aneurism of the Aorta or heart disease, the following pills act as a sedative: Digitalis 5.0, muriate of morph. 0·3, camphor 20. Forty pills to be given, one in the morning and at bed-time, slowly increasing the dose.—*Revue Medicale.*

Hypodermic Injections of tincture of iodine (¼ to ⅓ of a syringe full), in enlarged tonsils, and carbolic acid externally applied in acute rheumatism, are recommended.—*Memorab.*

NITRATE OF SILVER IN BED-SORES.—Betz regards nitrate of silver as the best remedy for bed-sores. Instead of making use, however, of lint dipped in the solution of luna caustic, he prescribes an ointment composed of five decigrammes of the nitrate of silver, fifteen grammes of lard, and thirty of wax; which he spreads on linen and applies to the sores, taking care that the piece is rather larger than the sore. This is repeated morning and evening.—*The Doctor, April* 1.

COD-LIVER OIL EMULSION.—Take of—

| | |
|---|---|
| Tragacanth. . . . . . . . | 8 parts. |
| Cold Water, . . . . . . . | 500 " |

Make into mucilage.

This, by being simply agitated, makes with cod-liver in all proportions a homogeneous mixture, which may be rendered more agreeable by the addition of 4 parts of alcohol with a little oil of bitter almonds and a trace of oil of cinnamon to every 30 parts. —*Journ de Pharm., April* 1872.

CHLOROFORM IN PUERPERAL CONVULSIONS—Chloroform is coming to be regarded as a most valuable remedy for the treatment of Puerperal Convulsions: several cases have been reported lately in the various medical journals, in which that treatment proved highly serviceable. In those cases in which we have had the opportunity of trying it, it has succeeded admirably; and we have, therefore, no hesitation in recommending it in all cases in which there is no contra-indication to its use.—*Canada Lancet.*

POWDER STOMACHIQUE.—Take of

| | |
|---|---|
| Powdered Rhubarb. . . . . . . | 3 grms. |
| Powdered Chalk, . . . . . . . | 3 " |
| Powdered Opium, . . . . . . | 25 centigr. |

Mix and divide into 12 packets. One powder half an hour after meals.—*L'Union Medicale.*

HOUSTON'S OPERATION.—Hæmorrhoids, connected with prolapsus, were successfully operated upon by the application of nitric acid some 30 years ago by Dr. Houston, of Dublin. The method found great favor at the time, but has been somewhat displaced by the ligature, and the section followed by the actual cautery. Billroth, of Vienna, has of late revived Houston's operation, and reports excellent results, especially in those cases where frequent and dangerous hæmorrhage had occurred.—*London Lancet.*

Abortive Treatment of Gonorrhœa.—M Ledeganck maintains in a pamphlet lately published at Brussells, that by cauterizing the fossa navicularis on the third day the disease can be arrested, as the inflammation has not yet extended further. Those who have much experience in the treatment of this complaint know that such a measure does not arrest the discharge, and that, by the application of the nitrate of silver, abscesses on either side of the frenum are likely to appear.—*London Lancet.*

Syrup of Chloroform.—Take of

| | |
|---|---|
| Pure Chloroform, - - - - - | 5 parts |
| Rectified Alcohol, - . - - - - | 24 " |
| Simple Syrup, - . - - - - | 300 " |

Mix the chloroform and alcohol and add the syrup and shake. Dose. A tablespoonful.—*L' Union Medicale.*

Dry Earth as a Surgical Dressing.—P. S. Conner, M. D., Surgeon to the Good Samaritan Hospital, Cincinnati, Ohio, (The Clinic, Sept. 7, 1872), has been using the earth dressing, as advocated by Dr. Addinell Hewson, of Philadelphia, in fourteen surgical cases, at the Good Samaritan Hospital, and has thus far been much pleased with the results of this treatment. He will continue to use it whenever he finds it practicable so to do; and from a trial of it, extending over a three months' period of time, he heartily recommends it to the use of those who have not tried it.

Dust-Powder for Infants.—Take of

| | |
|---|---|
| Starch, - - - - - - - | oz. j. |
| Oxide of Zinc, - - - - - | dr. ij. |

S. Use with a brush.

Cholera Remedy.—New Remedies for April, 1872, contains the following cholera prescription, a favorite one of Dr. H. Hartshorn, of Philadelphia: R—Chloroform, Tinct. Opium, Spts. Camphor, Spts. Ammonia Aromatic, aa f. dr. iss.; Creasote, gtt. iij.; Oil of cinnamon, gtt. viij.; Brandy, f. dr. ij. Mix. Dilute a teaspoonful with a wine-glass of water, and give two teaspoonfuls every five minutes, followed by a lump of ice.

Delfraisse Revulsive Liniment.—Take of

| | |
|---|---|
| Oil of Turpentine, - - - - - | 30 parts. |
| Tartar Emetic, - - - - - - | 4 " |

Mix. Frictions three or four times a day in rheumatic and neuralgic pains, until an eruption is induced.—*L' Union Pharmaceut.*

Injection of Air into the Uterus Causing Death.—Mention is made in the 'Gynæcological Journal,' Boston, for August, of a case of instantaneous death during the induction of criminal abortion by the injection of air into the uterus. The woman was quite dead when the physician arrived, and a Davidson syringe, which she had used, was lying beside her. An autopsy was made the following day. The uterus contained a fœtus of about six weeks. The membranes were unruptured, but were detached from the walls of the uterus in several places. A similar case occurred at St. Louis, some time ago. Another case was reported by Dr. Hitchcock, of Michigan, in the Trans. Am. Med. Association, 1864, p. 81. Death in this instance was supposed to have been caused either by the entrance of air into the circulation, or by shock. The *post-mortem* did not throw much light on the subject. —*Canada Lancet.*

Poisoning by Rumex Acetosa.—A case of death from poisoning by "sorrel" (Rumex Acetosa), is related by D. W. Brodnax, M. D., of Cameron, Milam county, Texas, in the July number of the Richmond and Louisville Medical Journal. The patient was a boy aged six years, who, in the language of a playmate, had eaten "a good deal of sheep sorrel." Although oxalic acid is said by writers on toxicology to be very speedy in its effects, in this case it had been twenty-four hours before his parents became sufficiently alarmed to call a physician, although he had been complaining, and his stomach very irritable, with frequent vomiting during that time.

Treatment of Burns and Scalds.—Dr. Montgomery, in the Pacific Medical and Surgical Journal, speaks highly of the efficacy of warm and soothing applications in the local treatment of burns and scalds. For that purpose he recommends poultices of slippery elm or linseed meal to be applied immediately, and covered with oiled silk. He records a number of cases in which this treatment was pursued, and with the most satisfactory result. It soothes the pain and excludes the air.

Antidote Against Carbolic Acid.—Th. Husemann recommends sugar-lime, prepared by dissolving 16 parts of white sugar in 40 parts of water, digesting with lime for three days, filtering and evaporating.

Chloral in Hydrophobia.—In the London Lancet, of April 20, I. D. Sainter, reports a case, which he considers to have been hydrophobia, cured by the free use of chloral.

TREATMENT OF GLEET.—Dr. Woodson, in the Kansas City Medical Jaurnal, recommends deep injections in the treatment of this affection. He uses a large sized catheter pierced at the curved end with small holes, for the space of 2½ or 3 inches, and having the eyelets closed.

This instrument being introduced, the injection is thrown in by a strong rubber syringe. The diseased parts can only be reached by these means. He also applies small blisters to the perineal portion of the urethra. The injections used are Tr. Iodine 1 drachm to the ounce of water, nitrate of silver 5 grs. to the ounce, or Monsel's solution (Ferri persulphas).—*Canada Lancet.*

TO STOP THE BLEEDING FROM LEECHES.—Make a ball of cotton about the size of a pea; put this pellet of cotton or lint upon the wound; press it down firmly; keep up the pressure for a quarter of an hour. Remove the finger cauteously, taking care to let the pellet remain.—*Druggists' Circular.*

HYPOPHOSPHITES IN THE TOOTHACHE OF PREGNANCY.—Dr. Sterling believes that the toothache so common in pregnancy results from the abstraction from the blood of the salts requisite for the construction of the bones of the fœtus, and accordingly recommends one and a half grains of hypophosphites of lime, soda, and magnesia, daily.—*Nashville Jour. Med.*

HYPERTROPHIED TONSILS.—NEW TREATMENT.—The application of fine needles of chromic acid to the tonsils causes notable shrinking of the parts, and is almost without pain or danger. By frequent application of this remedy the hypertrophy may be reduced to one-half its volume. Iodine dissolved in 100 parts of glycerine is also injected into the tonsil in some cases.—*Dr. Frœnkel, in the Berliner Klin' Woch.*

QUININE IN CHOLERA.—Professor Bolkin, in the cholera epidemic which took place in St. Petersburg, in 1871, made use of the sulphate of quinine, which he administered in doses of 20 to 30 centigrammes at a time, three or four times a day, and even more frequently if the substance was vomited; in many cases he also made use of a subcutaneous injection of the remedy. After using this remedy the mortality among his patients fell to 17.3.—*The Doctor, April* 1, 1872.

A SIMPLE POULTICE.—Apply thick wet cloths as dressing and cover with *waxed* paper. Greased paper answers this purpose pretty well also, and can always be readily obtained.

Fracture of Base of Skull.—A remarkable case of recovery after fracture of the base of the skull is reported in the Glasgow Medical Journal, for August, 1872, by Dr. Kelly, of Glasgow. The patient was 21 years of age. He was injured by the falling of a mass of coal, weighing about two hundred weight. Blood flowed from his nose, mouth, and left ear. The latter continued about two days, and was followed by total deafness, and the escape of watery fluid, which continued about 12 days. The quantity of fluid that escaped was estimated at about 14½ pints. Twelve weeks after the accident all the threatening symptoms had subsided, but sensation was deficient on the left side of the face and head, and the muscles paralyzed. The left ear was completely deaf, but his intelligence was unimpaired. The case is interesting, as showing that recovery may take place even in this usually fatal accident.—*Canada Lancet.*

Ulcer of Cornea and Dental Abscess.—That a diseased tooth may be an immediate cause of a corneal ulcer, or an active agent in producing its cure, Dr. C. E. Wright, of Indianapolis, Indiana, (Indianapolis Journal of Medicine, July, 1872), would admit. He publishes the case of a lady, aged 40, who had an indolent round ulcer on the corner of the right eye, and had been subject to violent paroxysms of toothache and neuralgic pains on right half of face and head for months; after proper ophthalmological treatment she was advised to consult her dentist, Dr. P. G. C. Hunt, who extracted the first upper molar on the right side, finding an abscess at the root. The same treatment of the eye was continued, and in about three days the eye was well.

Excision of the Hip-Joint for Morbus Coxarius.—T. Curtis, M. D., of Middleport, Ohio, (Medical and Surgical Reporter, Aug. 3, 1872), publishes a case of morbus coxarius in which life was prolonged by this operation, and believes if the patient had been seen two months earlier it would have been much better for final recovery. He warmly advocates excision after other means have had reasonable time and opportunity to arrest the disease without success, and where there are no very serious contra-indications.

Effect of Ether on Uterine Contractions.—At a late meeting of the Obstetrical Society of Boston, Dr. Abbott (Boston Medical and Surgical Journal, August 15, 1872), mentioned a case of labor, in which uterine contractions ceased after the full use of ether; although it has been said that anæsthetics in labor never interferes with the expulsive action of the uterus.

*Carrots versus Tœnia.*—Dr. Jos. Ganghofer details, in the Kansas City Medical Journal, the case of a boy, æt. 3½ years, who was suddenly seized with a craving for carrots, both cooked and raw. Persisting in this unnatural appetite, and being allowed to gratify it for six weeks, he passed, on the 12th of October, a *tœnia solium* of about eleven feet in length, body and head coming away at the same time. After this, the doctor being consulted, he found the little patient suffering from none of the symptoms of worms excepting a largely distended abdomen. The free use of carrots was recommended, and on the 24th of the same month, another worm was passed; this time a *tœnia lata*, 10 feet long, which came away entire, inclusive of the head. After the passing of this second specimen, the distention of the abdomen ceased, and the morbid craving for carrots was lost. A few weeks later, however, a number of links again appeared in the evacuation. The child having no longer any desire for carrots, a little kousso was given, and a few more links passed, which ended the difficulty. —*New Remedies.*

*Nitrate of Silver in Local Inflammations.*—Dr. George Crowell commends most highly the application of nitrate of silver in local inflammation, such as boils, carbuncles, testitis. In the latter disease he applies it in the following manner: The scrotum is made to present a tolerably smooth surface of skin over the swollen testicle, which surface is then wetted by means of a piece of lint previously dipped in water, and the solid nitrate of silver is then carefully and equally applied over the whole surface, suspensory bandages and rest being enjoined. In from two to six hours the pain has disappeared, and after this a gradual diminution of the swelling occurs. In boils and similar phlegmons he uses the solid stick or the strong solution (20 grs. to the drachm), as originally advised by Mr. Jno. Higginbottom.—*London Practitioner, February*, 1872.

*Recovery from Psoas Abscesses after Iodide of Potassium.*—In the Medical Archives for January, 1872, Dr. C. Du Hadway reports a case of chronic psoas abscess in which the ingestion of two drachms of iodide of potassium was followed by immediate recovery of the appetite and healing of the abscess in ten days.

*Chloral as a Local Application in Chancres.*—Dr. Francisco Accettella has been using chloral locally, with surprisingly good results, in the treatment of very old, obstinate chancres. He uses a solution of the strength of 1 to 4 for old cases; for less obstinate ones a weaker solution is employed.—*Revue de Therapeut. Medico-chirurgicale, April* 1, 1872.

*Epithelioma, treated by Carbolic Acid and Cured.*—In the Revue de Therapeutique Medico-Chirurg., of March 1st, is reported a case of epithelioma of the lip, by Dr. Forne, in which a cure was effected by the local application of crystalized carbolic acid to the sore.

Dr. Bois (Allgem. m. Centbl., 1870, 90 St.) recommends the use of pure carbolic acid in condylomata, which are attacked by the acid in all the thickness of their tissues, and then assumes a dullish white color; the neoplasm falls entirely after a single or a few applications of the remedy, without leaving behind any ulcer. This method of treatment, says the author, is preferable to any other. The acid may be used in a liquid form with a brush; it has no tendency to extend beyond the diseased parts. When carefully applied, it never causes any notable inflammation of the neighboring parts, nor any pain worth mentioning. It appears that this treatment is radical, for up to this time he has not noticed any relapse.—*The Doctor, March* 1.

*Treatment of Hæmoptysis by Atomized Perchloride of Iron.*—F. R. C. S., recommends strongly (The British Med. Jour. of Feb. 24), the use of liquor ferri perchloridi, one to four of water, with the atomizer in hæmoptysis, citing an instance of cancer of the lungs.

[The liquor ferri subsulphatis, U. S. P., seems to me to afford the most rational and successful method of treating hæmoptysis. It should be first used of the strength of thirty drops to the ounce, and the strength be increased if needed. Generally the iron salt is very well borne, and does not excite coughing. A saturated solution of alum is less efficient than the iron, but may be used when the latter excites irritation.—Ed. N. R.]—*New Remedies.*

*Aconite in Lichen and Prurigo.*—

R.—Ext. Aconite,
Ext. Taraxacum, - - - aa 1 gramme.

Make forty pills—one or two to be taken morning and evening. The itching soon ceases, and the eruption rapidly disappears.

*Effects of Bromide of Potassium.*—Dr. Julius Levy, of Berlin, writes that is bromide of potassium, in drach doses, three times daily, is continued for months, a series of boils will be apt to be produced. He says if some preparation of cinchona be given with the bromide, no boils or other evil sequelæ will arise.—*New York Medical Record.*

*Character and Treatment of Malignant Scrofulous Angina.*—Dr. Constantin Paul, in a long article published on the above subject in L'Union Medicale (number for July 23d), reviews the whole question of malignant scrofulous angina, as elucidated by the details of twenty cases which he has been able to observe. The chief points of the paper may be summed up thus: There does exist a malignant ulcerated angina of a scrofulous nature, the characters of which are now sufficiently recognized to enable us to form a correct diagnosis and to prevent us from pursuing an injurious mercurial (anti-syphilitic) treatment. Cod-liver oil and the general remedies used in scrofula are to be employed. Locally, tincture of iodine and perchloride of iron have not been very successful in Dr. C. Paul's hands, but he has found the acid nitrate of mercury to be of service.—*Lancet, August* 10, 1872.

*Injection for Gonorrhea.*—Dr. Reynolds (Proceedings College of Physicians and Surgeons, Louisville), mentioned that he had used the following injection with almost uniform success in a number of cases of gonorrhea that he had recently treated.

| | |
|---|---|
| R.—Balsam Copaiba, - - - - | dr. x. |
| Cryst. Carb. Soda, - - - - | dr. xx. |
| Water, - - - - - - | oz. xxx. |

To one part of this emulsion add three parts of water, and inject through a catheter carried into the urethra four or five inches as often as twice or three times a day. In chronic cases he used equal parts of water and the emulsion.

*Milk Fever.*—Dr. Scudder (Eclectic Medical Journal), writes: "Why should your patient suffer little or much from milk fever? when the administration of small doses of aconite, the second and third days will prevent it. If the breasts become hard and painful, add to the aconite, tinct. phytolacca, (green root). With these I have no fears of mammary inflammation or abscess, or the minor ills of caked breasts, or "ague in the breast."

*Excision of the Kidney.*—Dr. George A. Peters, it is stated (Medical Record, July 15, 1872), removed from a male patient at St. Luke's Hospital, the right kidney for abscess of that organ. The result is not given.

*To Prevent Stains from Tincture of Iodine.*—It is stated that a few drops of carbolic acid added to an ounce of tincture of iodine will prevent the unsightly stains left by the latter. The effect of the iodine is not impaired.

CEREBRO-SPINAL MENINGITIS. By E. Tefft, M. D., Topeka, Kansas.—In the August number of the Herald, hypodermic injections of morphia are recommended in cerebro-spinal meningitis, with ice to the back of the neck. The injections I have never used, and consequently have no personal experience in their operation. I have treated my last cases as I do acute rheumatism (and I believe this is the true nature of the disease.)

I gave large doses of quinine and Dover's powder in the commencement, alternated with iodo-bromide of calcium (Tilden's) accompanied by warm fomentations at the base of the brain and upper portion of the spine. Thus far this treatment has been attended with complete success.

I have just treated one of the most unpromising cases I ever saw with the best of results. The child had partial spasms every few minutes; pupils dilated and contracted alternately to the full extent; head thrown back, gasping for breath, screaming out with agony; urine scanty; extremely tender over the base of the brain and upper portion of spine; cold feet and hands, and pulse frequent, small and wiry.

I had but little hope of success, but treated it as above indicated, and it improved from day to day until it got perfectly well.

I do not know that the iodo-bromide of calcium is any better than other articles of the same class, but as it is the one I have been using in rheumatic affections more than any other, I used it in this case, and like its effects. I saw a report by a doctor in Illinois, who states that he has cured nearly every case by a similar treatment, and from my own experience I do not hesitate to recommend it to others.—*Leav. Med. Herald.*

BLENNORRHAGIC ORCHITIS.—Take of Crystalized nitrate of silver, - - - - - - 1 part.
Distilled water, - - - - 100 parts.

Dissolve. Saturate and compress with this solution, and maintain it constantly applied to the affected testicle. According to Dr. Marc. Girard this application does not cause pain, but simply a feeling of warmth. In about twenty-four hours the pain of the orchitis disappears, and in six days generally the cure is complete. —*L' Union Medicale.*

TREATMENT OF PHAGEDENIC CHANCRE.—Mr. Pollock, of St. George's Hospital, London, invariably treats this disease by from 15 to 20 drops of laudanum every four hours, combined in some cases with half a drachm of compound spirits of ammonia.—*London Lancet,* Dec. 16, 1871.

THE TREAMENT OF HYPERÆSTHESIA OF THE VULVA AND VAGINA. M. Gueneau de Mussy (Gaz. des Hopitaux, June, 1871) is strongly opposed to the treatment adopted in vaginismus, by Sims, and he thinks that the wise combination of therapeutic agents, together, if need be, with progressive or sudden dilatation, will often render resort to deep incisions unnecessary. He has often obtained the most happy results from the action of vaginal suppositories:

| | |
|---|---|
| Cacao butter, - - - - - - | 2 gram. |
| Bromide of Potassium, - - - - | 30 centigr. |
| Extract of Belladonna, - - - - | 10 " |

This suppository he introduces every night, and its use is continued for two or three weeks.—*Birmingham Med. Rev.*, Jan. '72.

CONCENTRATED EMULSION OF ALMONDS. (M. REYNOLLS.) Take of—

| | |
|---|---|
| Blanched sweet almonds, | |
| Sugar, | |
| Glycerine, each - - - - | 30 parts. |
| Powdered gum arabic - - - - | 4 parts. |
| Water - - - - - - | 60 parts. |

Reduce to a uniform paste, strain and evaporate at a temperature not exceeding 60 deg. F. to the condition of a nearly a solid extract. In order to prepare at any time, from this concentrated emulsion, the ordinary almond emulsion, add to two drachms of it enough simple water, or (more elegant) water of orange-flowers, to make an ounce of fluid.—*Jour. de Pharm. et de Chim.*, April, 1872.

BREAD MADE WITH SEA-WATER.—M. Rabuteau calls attention to the importance of this article. In the first place it is very pleasant eating, also increasing the appetite and stimulating digestion. On board ship bread so prepared has been found very conducive to the preservation of health during long voyages. It also exerts important medicinal effects, especially in dyspepsia. In phthisis and in scrofula the author states it is a powerful adjuvant. —*Med. Times and Gaz.*, Feb. 3, 1872, from *L' Union Med.*, Jan. 6, 1872.

LOCAL APPLICATION OF SULPHATE OF IRON IN PHLEGMASIA DOLENS.—In the Boston Med. and Surg. Jour., Dr. Crighton strongly advocates, in recent cases of phlegmasia dolens, the employment of a lotion of from 20 to 30 grains of sulphate of iron to each ounce of water, to be applied as hot as the patient can bear it.

SENECA ROOT—AN OLD REMEDY REVIVED.—Dr. Thomas Barrow, of Baltimore, (Phil. Med. Reporter,) is enthusiastic in praise of the root of Polygala Senega in chronic bronchitis, and states a number of cases of several years' standing, which were cured by it in a very short time. He prepares it in various ways to suit the circumstances of each case. One of his favorite prescriptions is this: R. Syrupi senegæ f oz. iiss.; mist. glycyr. comp. f dr. iv.; Tart. ant. et pot. gr. j.; sulph. morph. gr. iij. M. Dose, half a teaspoonful every four hours.—*Pacific Med. Journal.*

BROWN WATER OF WARLOMONT.—Take of—

| | |
|---|---|
| Borax, - - - - - - | 10 parts. |
| Extract of hyoscyamus, - - - | 5 " |
| Decoction of mallow roots, - - - | 180 " |

Mix.

Employed in ucerative keratitis, catarrhal ophthalmia, and other acute affections of the eye. To apply it, a compress should be wet with it (after shaking) and firmly bound on the eye. The compress should be kept wet for half an hour and then removed for two hours, at the end of which time it should be reapplied as before.—*Jour. de Pharm. et de Chim.*, April, 1872.

NOCTURNAL INCONTINENCE TREATED BY CHLORAL.–Dr. Girolamo Leonardi (L'Ippocratico, 10th July,) has found chloral useful even in adults long affected with nocturnal incontinence of urine, in the dose of one gramme dissolved in sixty grammes of water at bedtime. The patient is not to drink much at suppper. He mentions one case of rapid cure in a young scrofulous male, aged 24, and another in a boy, aged 7. The cure was without delay.—*Doctor.*

DANGER FROM GUTTA-PERCHA CATHETERS.—So many cases have occurred of the retention of fragments of gutta-percha catheters in the bladder, that surgeons are discarding such catheters entirely from use. They sometimes become brittle and crack like glass; or they soften when left in the urethra, and portions are retained in the bladder.

A NEW STYPTIC.—Collodion, 100 parts; carbolic acid, 10 parts; tannin (Pelouse's), 5 parts; benzoic acid (from gum), 5 parts. Mix ingredients in the order above written, and agitate until solution is effected. This preparation has a brown color, and leaves on evaporation a strong adherent pellicle. It instantly coagulates blood, forming a consistent clot, and a wound rapidly cicatrizes under its protection.—*Med. and Surg. Jour.*

Elixir Quiniæ Ferri et Strychniæ Phosphatis.

| | | |
|---|---|---|
| R.—Quiniæ, | - - - - - - | gr. xxx. |
| Ferri pyrophosphatis, | - - - | gr lx. |
| Strychniæ, | - - - - - | gr. j. |
| Acid citrici, | - - - - - - | gr. xxx. |
| Alcoholis, | - - - - - | f oz ij. |
| Syrupi | - - - - - - | f oz iss. |
| Aquæ aurant. flor. | - - - - | f oz iss. |
| Glycerinæ, | - - - - - - | f oz ij. |
| Aquæ destill. q. s. ad. | - - - | f oz viiss. |
| Aquæ ammon, | - - - - - | q. s. |

Dissolve the iron in f. oz. ss. of water; mix the syrup, glycerine, and orange-flower water, and add to the solution of iron; then add oz. ss. of alcohol. Dissolve the quinia, with 5 gr. citric acid, in oz. j. water and oz. j. alcohol, by the aid of heat; then mix with the iron and syrup solution. Dissolve the strychnia in the remainder of the alcohol, and add to the other solution; then add the remainder of the citric acid, in powder, with enough liquid ammonia, until it becomes clear, using a little heat after the acid is added. The quinia solution must be of the same temperature as the iron when added, also with the others when added. This gives a beautiful straw-colored elixir, representing about 1 gr. sulphate of quinia. 1 gr. pyrophosphate of iron, and one-tenth gr. strychnia in the fluid drachm.—Chas. Shivers, Jr., *Amer. Jour. of Pharm.*

External Use of Fuming Nitric Acid.—Professor Patruban (Memorabilien, June, 1872), says that this heroic caustic is indicated on account of its rapidity and drying-up of the tissues. It stops all effusion of blood, and leaves good scars. He uses it for warts and other protuberances of the skin, as condylomata, epulis, hæmorrhoids, and tumors of the nature of nævus.—*Doctor.*

Ointment for Piles.—M. F. Guym, of the Necker Hospital, Paris, prescribes in painful hæmorrhoids an ointment compound of one part of extract of belladonna, two of extract of rhatamy, and fifteen of lard.—*Doctor.*

Morphia in Diabetes.—Dr. Comfort (Canada Lancet), at a meeting of the Ontario Medical Society, mentioned a case of diabetes treated by small doses of morphia at regular intervals, presevered with for about four months, and terminating very satisfactorily in convalescence, though the quantity of urine passed in 24 hours had reached eight quarts.

Emmenagogue Formula.—Dr. Theophilus Parvin, says: A good emmenagogue pill in anæmic amenorrhœa is composed of equal parts of dried sulphate of iron, white turpentine, and aloes. The pill may weigh two or three grains, and may be given twice or thrice daily, regulating the frequency of repetition and the weight of the pill by the condition of the bowels.

Another efficient emmenagogue pill may be made of equal quantities of *rue*, *savine* and *ergotine*, and half as much either of aloes or of gamboge. The pill may weigh from three to four grains, and two or three may be given three times a day. *Apiol*, the active principle of parsley, is not held in such high esteem in this country as it is in some parts of Europe; partly because it has been so little tried, but chiefly because that which is generally found in our drug stores is either entirely factitious or else grossly adulterated. Undoubtedly this medicine is one of the safest and most certain of that very uncertain class of medicines, called emmenagogues.

*Faradization*—the positive electrode passed into the uterine cavity, the negative applied to the hypogastrium—gives oftentimes a very prompt success in inducing a sanguineous discharge from the uterus; but in order that such result should follow, this means should be used only at a time when the other phenomena of menstruation manifest themselves, the flow only wanting.—*Am. Prac'r.*

Grafting with Rabbit Skin.—There have been reported to the Paris Academy of Sciences three cases of ulcers in the human subject healed by grafting upon them cutaneous particles taken from the rabbit. M. Larrey was so pleased with the idea that he proposed entering the Report for the prize. Whether the new surface produced a growth of rabbits' fur, we are not informed. But why not? And if so, why not put the new practice to profit by substituting for the rabbit the Angora goat or some other variety which will preduce a valuable wool? Baron Munchauson did something of this kind, if our memory serves us, with his horse. There are some thousands of vagabonds and convicts in California, who might be made valuable to the State, or at least who would pay for their keeping, if, by the aid of delicate surgery, they were set to raising wool.—*Pacific Med. Journal.*

Hydrocele and cysts can be treated by a new method of Monod. He takes out some fluid and injects the same quantity of alcohol. The injections repeated every fourth day, cause no pain nor inflammation. Resorption is effected by altering the fluid.—*Indiana Med. Journal.*

Action of Soda.—Dr. Scudder, (Eclectic Med. Journal,) remarks: I have just had a case in hand that markedly illustrated the specific action of Soda. A young miss of fifteen years, has suffered from a pustular eruption making its appearance every Summer in June and continuing until October. It originated in poisoning by Rhus, and has been very intractable and painful, and has left large scars on the body.

This year it commenced as usual, in a very severe form, especially upon the face and neck, and the mother fearing deformity applied to me for treatment, though medicines and local applications had not been of the least benefit. What now should I give her, or how should I be guided in selecting a remedy?

I gave the patient a careful examination, and the only pronounced symptom was a broad *pallid* tongue. It was the indication for Alkalies, and I accordinngly prescribed Bicarbonate of Soda, as a general remedy. In ten days the disease was at an end, the amendment commenceing within twenty-four hours.

I should have used the remedy locally, as I do in recent poisoning by Rhus, but the skin was so irritable that she could not bear the weakest solution.

Liquid Shampoo.—Take Bay rum, - - 2½ pints.

Water, - - - - - - ½ pint.

Glycerine, - - - - - 1 ounce.

Tinct. of cantharides, - - - 2 drachms.

Carbonate of ammonia, - - 2 "

Borax, - - - - - ½ ounce.

Dissolve the salts in water and add the other constituents gradually.—*Druggists' Circular.*

Cure for Sleep-Wakling.—This troublesome habit oftentimes resists all the common-place plans to break it up. When such is the case, Dr. Pelizzo (in the Abeille Medicale) recommends the use of bromide of potassium. We tried it in the case of a girl who would get up in her sleep, walk about her room, etc., etc. About 30 grains daily (morning and evening) effectually cured. It is also of good service in the agitated and restless sleep of young children.—*Med. and Surg. Reporter.*

*Migraine.*—Dr. Gauchet recommends the following pills in migraine during the monthly discharge: chinin. sulph. 3 grammes; pulv. digital. 1, 5 grammes, ft. pil. No. 30, S., one at bedtime.—*Bul. gen. dc Therap.*

PRUNIN—is the active principle of *prunus virginiana.*

*Properties.*—Stimulant, tonic, expectorant, and, in large doses, sedative.

*Employment.*—Coughs, colds, incipient phthisis, dyspepsia, hectic fever, debility, scrofula.

In diseases arising from disturbances of the heart it is almost specific; also, in chronic bronchitis, depending upon valvular disease of the chambers of the heart.

This is a valuable stimulant, tonic and expectorant in small repeated doses, and an arterial sedative in large doses; valuable in cases of enfeebled digestion, especially in the convalescing stages of pneumonia, fevers and other acute diseases; incipient phthisis and æsthenic forms of dyspepsia. Appropriate in hectic fever, giving tone to the cutaneous capillary structure, and restraining the tendency to colliquative sweats. It is also useful in hooping cough, irritability of the nervous system, and in scrofula, amenorrhœa, dysmenorrhœa, leucorrhœa, etc. Its sedative properties are said to be due to the hydrocyanic acid which it yields.

As a lotion, it is frequently of good service in the treatment of ill-conditioned ulcers.

*Dose.*—From two to five grains, thrice daily.

OIL OF STILLINGIA.—Derived from *stillingia sylvatica*, or queen's root. This oil is reckoned too acrid for internal use, but when administered possesses nearly the same properties as the active principle of stillingia. Nevertheless, we have found it of great value when administered, internally, in cases of bronchitis, laryngitis, defective menstruation, chronic gleet, leucorrhœa, etc.

*Dose.*—One drop in water or upon sugar.

It overcomes spasm and difficulty of respiration, and facilitates expectoration; and, hence, is useful in asthma, hooping cough and similar affections.

For the relief of asthma it may be combined with oil of lobelia. Used externally, the oil is an invaluable stimulant, counter-irritant and relaxant.

NEW REAGENT FOR BLOOD.—According to H. Struve, the coloring matter of the blood is best precipitated by tannin, the liquid being first rendered strongly alkaline by ammonia or caustic potassa. After the addition of the tannin, the liquid is rendered acid by a sufficient quantity of acetic acid; the precipitated tannate of hæmatin is collected, washed and dried, and, on treatment with ammonium chloride and glacial acetic acid, yields crystals of hæmin.—*Detroit Rev. of Med.*

*Use of Diluted Pyroligneous Acid as a Gargle.*.—Put a teaspoonful of the acid obtained from the shops into a wine-glass of water and direct the patient to gargle the throat frequently with it.

In the sore throat caused by exposure, so common throughout the country, it generally relieves the soreness and stiffness felt in swallowing very promptly.

In chronic inflammation, with or without ulceration, of the throat, I have found it a very valuable remedy.

In the sore throat of Scarlatina it has generally afforded a very prompt amelioration of this symptom of the disease.

In several cases of habitual tonsilitis, by using this gargle freely at the commencement of the disease, I have been able to arrest the progress of the inflammation and secure a resolution.

Its use is not unpleasant; it is safe even if used for hours continuously, and has an additional advantage in removing the fœtor of the breath.

*Secondary Syphilis.*—Dr. D. W. Yandell (Proceedings of College of Physicians and Surgeons), reported a case of syphilis sent to him from a neighboring town, where the disease had been contracted by repeated and prolonged kissing a young woman who had syphilitic roseola, with mucous patches on the inside of the cheeks and lips. There were mucous patches in the mouth, intense redness of the throat, and a well-marked rash upon the trunk of the patient; but no trace whatever of chancre or inguinal induration had ever been present, according to the testimony of the gentleman, who was perfectly reliable.

*Hydrocele.*—Mr. Bradley, of Manchester, recommends after the liquid of hydrocele is withdrawn, that the testicle of the afflicted side should be tightly strapped with soap plaster. In four cases in which this was done, complete obliteration of the vaginal sac took place, with a consequent radical cure.—*The British Medical Journal, June* 1, 1872.

*A New Adhesive Substance.*—Dr. Mellez proposes the following as a substitute for collodion. Dissolve shell-lac in alcohol with a moderate heat, in such proportions as to give it the consistence of jelly. This is simply spread upon suitable cloth. It is not so transparent as collodion, nor does it dry so rapidly.

*Morphia and Chloral.*—Dr. R. H. Fisher speaks very highly of the combined use of morphia and chloral for relief of pain.—*Medical News, July*, 1872.

THE AGENCY OF THE MIND IN DISEASE.—Dr. Flint says, in the American Practitioner: "Physicians, sanitarians and moralists have of late had much to say respecting the evils of over-exertion of the intellect and of mental strain; and there has been much occasion for speaking of these sources of injury to mind and body in our country especially during the past few years. But there is another aspect of the etiology of morbid mental conditions concerning which much less has been said, namely, deficient exercise of the intellectual powers, or insufficient activitv of the mind as a source of morbific agences. Observation will, I think, show the evils of both body and mind originate quite as often in a want of the proper action of the intellectual and moral faculties as in their over use or excitation. Occupations which employ the intellect are likely to prevent inordinate attention to the bodily functions, and herein their influence is prophylactic. Abundant illustrations of the evils of deficient activity of the mind are to be found among those who, under the delusive expectation of enjoying leisure and rest, have relinquished pursuits which involved a habitual exercise of the mental faculties.

*Modification of Pettenkofer's Test for Biliary Acids.*—M. Strasburg, of Bremen, adds to the urine suspected to contain biliary acids a little cane sugar, and dips into the liquid a slip of filtering paper. The slip is then dried, and a drop of pure concentrated sulphuric acid is applied to it with a glass rod. If biliary acids are present, even in the proportion of 3:100,000, the characteristic violet coloration makes its appearance after a few seconds.—*Detroit Rev. of Medicine.*

HYDROCHLORATE OF AMMONIA.—Dr. Spencer Thompson says, in the British Medical Journal, that he has found this one of the most efficient remedies in portal dropsey, in scruple or half drachm doses, tolerably diluted, every six or eight hours.

OIL OF STAPHYSAGRIA.—This oil has been obtained by distillation from the seeds of the staphysagria, and is more active than the seeds themselves in destroying pediculi.

The oil amounts to as much as half of the weight of the seeds. Ether also extracts the oil. Digesting the crushed seeds in hot lard makes a splendid ointment for certain skin diseases.

HOW CUNDURANGO CURES CANCER.—Mrs. Mathews, the mother of Vice President Colfax, died recently from the cancer which was cured a year ago by Cundurango.—*Pacific Med. Jour.*

*New Method of Nourishing by the Rectum.*—Dr. W. O. Leube states that he, after long experience, has perfected a method of feeding by the rectum much superior to the old plans, so that he has been able to support a patient for a month, to whom no food whatever was given by the mouth. The injection causes no pain, but rather a general feeling of bien-faisance. The preparation is made as follows: The pancreas of swine or cattle is carefully cleaned of fat, and 50 to 100 grammes thereof cut into very small pieces. In like manner 150–300 grammes of beef are prepared. Both substances are then put in a dish with about 50 to 150 cc. of lukewarm water, and stirred into a thick paste, and drawn in a clyster-pipe with wide opening. In many cases to the mixture from 25 to 50 grammes of fat may be added, also at times some starch. An hour before using this clyster one of pure water should be administered to clean out the small intestines.—*Deutsches Archiv fnr klinische Medicin*, March 15, 1872.

*Antacid for Heartburn.*—

| | |
|---|---|
| R.—Potassæ Subcarbonat, - - - | aa dr. ij. |
| Spirit. Lavand. Comp., - - - | |
| Sacchar. Alb., - - - - - | dr. j. |
| Tinct. Opii., - - - - - | gtt. xxx. |
| Aquæ, - - - - - - - | oz. iii ss. |

M. S.—Tablespoonful as required.—Dr. B. S. Thompson, *in American Practitioner.*

*Cotton Wool as a Dressing.*—Dr. Wm. Warren Greene commends cotton wool very highly as a dressing for wounds. He uses it in a way similar to that or M. Guerin.—*Boston Med. and Surg. Journal*, May, 1872.

*Enlaryed Testicle.*—Dr. D. Hayes Agnew used the following for indolent enlargement of the testicle, supposed to be strumous.

The man was ordered to take oz. ss. ol. morr., with gt. xv. syr. ferri iodidi after each meal; also, arsen. iodidi gr. 1-18 t. d. Locally to apply.

R.—Ext. Belladonna,
Ung. Hydrarg.,
" Iodine,
" Adipis, - - aa dr. ij.—M.

If the ointment irritates the skin, it may be further diluted.—*Med. and Surg. Reporter.*

*Urea in Bile.*—Popp finds that urea is a constant constituent of bile. The bile of the pig seems to contain it in larger quantity than that of the ox.

*Emulsion of Turpentine.*—I. Winchele Forbes highly commends (American Journal of Pharmacy) the following method:

First. Pour the turpentine into a two-ounce vial, and shaking so as to coat the inside of the vial with a film of turpentine; this is to prevent the action of the moisture usually present.

Secondly. I add one scruple powdered acacia, and mix thoroughly with the oil.

Lastly. Half a fluid ounce of water is added, and the whole is well shaken. A perfect emulsion is the result, requiring less time for its preparation than to read the foregoing directions. The bottle may then be filled up with mucilage, or, according to my experience, a better product is obtained with water simply.

The deviation from the letter of the law in regard to the gum strength of the emulsion needs no apology to the practical pharmacist, as the sole object in view is to emulse the oil, and it will be found that ten grains to the fluid ounce of emulsion will afford a product superior in all respects (especially in fluidity), to one containing more gum, and more nearly approaching the peculiar characteristics of that most perfect of all emulsions—cow's milk.

*Formula for Dyspepsia of Debauchees.*—Dr. James R. Wood's formula is commended by Dr. B. S. Thompson (American Practitioner, July, 1872). It is as follows:

| | |
|---|---|
| R.—Tinct. Valerian Aromat., - - - | } aa oz. ij. |
| Lupulin, - - - - - - | } |
| Tinct. Cardamom, - - - - - | oz. ss. |

M. S.—A teaspoonful as required.

*Carbolic Acid in Whooping-Cough.*—Dr. C. Glen Bott has found carbolic acid to have wonderful power in arresting whooping-cough. He gives $\frac{1}{24}$ to $\frac{1}{36}$ of a drop freely diluted with water every four hours to a child eight years old, or in some cases $\frac{1}{8}$ of a drop three times a day to a child four years old.—*Medical Times and Gazette*, June 29, 1872.

*Oxalate of Potash in Peritonitis.*—"Two cases of peritonitis with purulent effusion, the pus discharging itself by the umbilicus, have been reported this year to the Medico-Chirurgical Society of Liege, as resulting successfully. Other cases have also been reported, in which small doses of oxalate of potash in mucilage were given every hour, with the best results, the patients being perfetly and speedily restored to health."

*Acid of the Tomato.*—According to S. D. McElhenie (Journal of Pharmacy), the acids of the tomato are citric, oxalic, malic, and tartaric in very small quantity.

*Sick Headache.*—Dr. Nunneley advises, in hemicrania or nervous sick headache, drachm doses of the aromatic spirits of ammonia with ten-grain doses of the chloride of ammonium, to be given as soon as the attack begins, and to be repeated every twenty minutes or half hour for two, three or four doses, and then at longer intervals; or tincture of nux vomica, in doses of two minims every quarter of an hour for two or three hours, afterwards at longer intervals. A few doses of one or other of these medicines may cut short a commencing sick headache. He also advises hydrate of chloral at bed-time, and bathing the head with hot or cold water, according to the patient's wish.

Dr C. Allbutt advises bromide of potassium in sick headache (neuralgia), especially when in connection with uterine or ovarian disease; he considers hemicrania to be a neuralgia of the trigeminal nerves, and advises, in order to cut short an attack, twenty to sixty grains of nitrate of ammonia; as tonics, he gives arsenic the first, and strychnine the second place, and he advises the daily or tri-weekly use of the continuous current.

Dr. W. S. D. Williams states that the use of tincture or extract of cannabis indica daily during the intervals has proved very successful.

*Cholera Infantum.*—Dr. Scudder, (Eclectic Medical Journal), speaking of having employed various methods of treatment unsuccessfully, remarks: Then came the treatment by aconite alone, with quinine inunction, when I was almost ready to cry *Eureka.* To this was soon added nux vomica to stimulate the sympathetic, and ipecac as a special remedy for the disease of mucus membranes, and I felt satisfied that I had a rational treatment for cholera infantum.

For eight years it has been a success, in the treatment of hundreds of cases in my own practice, and in thousands of cases in the practice of other physicians, since it was published. And to-day, I feel that I could risk the entire subject of "Specific Medication" on this alone.

*The "Rhubarb Bitters."*—

℞.—Tinct. Rhei Dulc.
Tinct. Cinchon. Comp.
Tinct. Gentain Comp., - - aa. dr. vj.
Tinct. Leptandrin,
Tinct. Nux Vomica, - - - aa. dr. j.
Water, enough to make - - oz. vj.—M.

Dose, a tablespoonful three times a day. Tonic, alterative, stomachic, carminative, laxative.

